# Postoperative parathyroid hormone (PTH) is equivalent to perioperative PTH drop in predicting postsurgical hypoparathyroidism

**DOI:** 10.1308/rcsann.2024.0001

**Published:** 2024-03-13

**Authors:** M Hashem, CB Lim, SP Balasubramanian

**Affiliations:** ^1^ Sheffield Teaching Hospitals NHS Foundation Trust, UK; ^2^ University of Sheffield, UK

**Keywords:** Thyroid surgery, Postsurgical hypoparathyroidism, Parathyroid hormone, hypocalcaemia

## Abstract

**Introduction:**

Postoperative surgical hypoparathyroidism (PoSH) following thyroid surgery is an established complication. Several predictive factors have been identified including perioperative parathyroid hormone (PTH) levels. The aim of the study is to compare the drop in perioperative PTH to postoperative day 1 PTH in predicting hypocalcaemia and hypoparathyroidism.

**Methods:**

Records of consecutive patients who had either total or completion thyroidectomy with or without central neck dissection in a 3-year period in a single thyroid surgery unit were accessed to retrieve data on demographics, pathology, surgery, perioperative biochemistry and management.

**Results:**

Of 295 included patients, there were 227 (76.9%) females. Forty-five (15.3%) had completion thyroidectomy, and the rest had total thyroidectomy. Seventy-eight (26.4%) had concomitant central neck dissection. Sixty-four (21.7%) had hypocalcaemia on the day after surgery. Hypoparathyroidism persisted in 10.5% of patients at 6 months. Both day 1 PTH and a drop in PTH predicted day 1 hypocalcaemia (*p* < 0.001) and 6-month hypoparathyroidism (*p* < 0.001). The area under the receiver operating characteristic (ROC) curves for day 1 PTH and drop in PTH for the prediction of day 1 hypocalcaemia (0.729 vs 0.726, respectively) and for 6-month hypoparathyroidism (0.964 vs 0.958, respectively) were similar, albeit slightly better for day 1 PTH.

**Conclusions:**

Day 1 PTH is equivalent to (if not better than) drop in PTH in predicting short- and long-term PoSH. Preoperative PTH measurements may not be needed in the detection and/or management of PoSH after thyroid surgery.

## Introduction

Postoperative surgical hypocalcaemia and/or hypoparathyroidism (PoSH) is a common postoperative complication after thyroid surgery, with reported incidence varying between 0 and 46%.^1,2^ Although the variation in incidence may relate to underlying thyroid pathology, extent of surgery, surgical techniques and experience, a significant component of the variation may be caused by the wide variation in the definition of this condition.^3^ The British Association of Endocrine and Thyroid Surgeons (BAETS) define transient hypocalcaemia as an adjusted calcium level below 2.10 mmol/L^3^ on the day after surgery, and long-term or persistent hypoparathyroidism as a condition whereby a patient is still on calcium and/or vitamin D supplements 6 months after thyroid surgery.^4^ Postoperative hypoparathyroidism can be caused by direct damage to or devascularisation of the parathyroid gland(s), obstruction to its venous drainage or accidental removal during operation.^5^ Occasionally, hypocalcaemia can occur independent of parathyroid hormone (PTH) and this can be caused by hungry bone syndrome in cases of Graves’ disease or intraoperative haemodilution.^6–8^

Risk factors for PoSH include preoperative hypocalcaemia, low vitamin D, inability to visualise parathyroid glands intraoperatively, need for auto-transplantation of parathyroid glands, central neck dissection, re-do operation, large or retrosternal goitre or other patient-related factors such as female sex and Graves’ disease.^1^

PoSH is associated with both short-term and long-term morbidity and mortality. In the short term, patients with PoSH need calcium and/or vitamin D supplements, closer monitoring and have a longer stay in hospital and a higher risk of readmission to hospital after thyroid surgery.^9^ In the long term, there is a higher risk of conditions including depression and affective disorders, hospitalisation relating to infections and seizures, renal complications, impaired quality of life and potentially higher mortality.^10–13^

Early detection and appropriate management of PoSH is important in reducing morbidity. In this unit, previous studies of PoSH after thyroid surgery have resulted in the development of a protocol based on calcium and PTH measurements on the first postoperative day.^1,14^ This protocol is currently used in the detection and management of hypocalcaemia. This has helped to reduce significant symptomatic hypocalcaemia and rates of readmission.^14^ Other studies have also reinforced the value of a formal protocol utilising either calcium, PTH or both variables in the early detection and treatment of this complication.^15–20^

It has been suggested that the decline in PTH values after surgery may be a better predictor of hypocalcaemia compared with postoperative values alone.^21^ However, this needs additional blood tests and a clear definition of the extent of the fall in PTH that would be the optimum predictor of hypocalcaemia. In a study of 176 patients who underwent total thyroidectomy, the decline in PTH after surgery was a reliable predictor of transient hypocalcemia, and a 71% reduction was proposed as the threshold with the highest sensitivity and specificity.^22^ Another study from Turkey of 100 patients undergoing total thyroidectomy proposed a drop of intact parathyroid hormone (iPTH) of >85% to be a good predictor of symptoms of hypocalcaemia; however, the postoperative PTH at 1h was equivalent to PTH decline.^17^

The aim of this study was to evaluate drop in PTH as a predictor for PoSH and compare it with postoperative PTH alone in the detection of postsurgical hypoparathyroidism.

## Methods

This was a retrospective cohort study of patients who underwent either total or completion thyroidectomy over a 3-year period (January 2016 to December 2018) in a single tertiary endocrine surgery unit. Indications for surgery included thyroid nodules suspected or confirmed to be malignant, nodules with compressive symptoms and hyperthyroidism. Patients who had concomitant central with or without selective neck dissection were also included. Patients who had hemithyroidectomy or concomitant planned parathyroidectomy were excluded. All operations were either performed or supervised by one of two consultant endocrine surgeons.

Demographics, indications for surgery, details of surgery, perioperative biochemistry, histopathology, occurrence and management of calcium-related complications were retrieved from electronic patient records. Perioperative biochemistry included corrected serum calcium, vitamin D, thyroid function tests and PTH levels.

The outcomes studied were the occurrence of transient hypocalcaemia and long-term hypoparathyroidism, defined in accordance with the BAETS guidance, as described previously. The normal range for PTH was 1.6–6.9pmol/L. The PTH level on the morning after surgery was recorded. The preoperative PTH level was chosen as the last available result over the last 6 months, and the drop in PTH was recorded as the percentage reduction in PTH from the preoperative value: [(preoperative − first postoperative PTH/preoperative PTH) × 100].

### Statistical analysis

All data were collected by one author in an Excel spreadsheet and the outcome data were validated by a second author. Analysis was done using IBM SPSS statistics version 26. Descriptive analyses were done using frequencies and percentages for categorical data. For continuous data that were not normally distributed, median and interquartile range (IQR) was used. The association between categorical variables was testing using the chi-squared test with Yates’ correction and the Fisher’s exact test, where appropriate. The association between continuous variables that were not normally distributed (such as age) and patient groups was tested using the Mann–Whitney *U* test. The relationship between PTH and the outcomes was tested using logistic regression and receiver operating characteristic (ROC) curve. Results were regarded statistically significant if the *p*-value was <0.05.

Because this was a service improvement project, individual consent from patients or approval from the ethics committee were not considered necessary. The study was registered with the clinical effectiveness unit of Sheffield Teaching Hospitals NHS Foundation Trust (reference no. 9331). All the data collected were initially stored in password-protected National Health Service computers and the data were anonymised prior to analysis and reporting.

## Results

The study included 295 patients (76.9% females, 23.1% males) with a median (range) age of 49 (16–88) years. The most common preoperative diagnosis was Graves’ disease (39.3%) followed by suspected/confirmed thyroid cancer in 36.9%. Other diagnoses included thyroid nodule(s) and hyperactive thyroid other than Graves’s disease (21.7% and 2%, respectively).

Total thyroidectomy was done in most patients (84.7%) with the remainder (15.3%) undergoing completion thyroidectomy. Table 1 demonstrates the number of patients undergoing central neck dissection and its association with the extent of thyroid surgery.

**Table 1:** Extent of thyroid surgery and neck dissection in the cohort studied

	Central neck dissection	
	Yes	No
Total thyroidectomy (*n* = 250)	53 (21.2)	197 (78.8)
Completion thyroidectomy (*n* = 45)	25 (55.6)	20 (44.4)

Values in parentheses are percentages

On the day after surgery, 64 patients (21.7%) were found to be hypocalcaemic. However, 100 patients (33.9) were discharged on calcium or vitamin D supplements. This figure dropped after 6 months to 31 patients (10.5%). One patient was excluded because of missing data. Table 2 shows the proportion of temporary and long-term PoSH in different groups of patients classified into various patient-, provider- and surgery-related factors. Day one hypocalcaemia (adjusted calcium of <2.1mmol/L) was associated with low PTH on the first postoperative day (*p* < 0.001) and the drop in perioperative PTH (*p* < 0.001). In addition, PoSH at 6 months was also associated with low PTH on the first postoperative day (*p* < 0.001) and drop in perioperative PTH (*p* < 0.001).

**Table 2:** Temporary hypocalcaemia and long-term hypoparathyroidism rates in different subsets of patients

Variable	Postoperative hypocalcaemia		*p-*value	Long-term hypoparathyroidism		*p-*value
	No	Yes		No	Yes	
Sex						
Male	60 (88.2)	8 (11.8)	**0.036** ^a^	62 (92.5)	5 (7.5)	0.479^a^
Female	171 (75.3)	56 (24.7)		201 (88.5)	26 (11.5)	
Median (IQR) age at surgery	50 (35–64)	46 (31–54)	**0.034** ^b^	49 (34–61)	47 (30–54)	0.264^b^
Thyroid surgery						
Total thyroidectomy	194 (77.6)	56 (22.4)	0.620^a^	223 (89.6)	26 (10.4)	1.0^a^
Completion thyroidectomy	37 (82.2)	8 (17.8)		40 (88.9)	5 (11.1)	
Central neck dissection						
Yes	57 (73.1)	21 (26.9)	0.252^a^	65 (84.4)	12 (15.6)	0.144^a^
No	174 (80.2)	43 (19.8)		198 (91.2)	19 (8.8)	
Consultant						
A	126 (82.4)	27 (17.6)	0.107^a^	143 (94.1)	9 (5.9)	**0.013** ^a^
B	105 (73.9)	37 (26.1)		120 (84.5)	22 (15.5)	
Number of parathyroids seen at surgery						
0	13 (92.9)	1 (7.1)	0.097^c^	11 (78.6)	3 (21.4)	0.358^c^
1	41 (87.2)	6 (12.8)		44 (93.6)	3 (6.4)	
2	94 (78.3)	26 (21.7)		106 (89.1)	13 (10.9)	
3	56 (76.7)	17 (23.3)		64 (87.7)	9 (12.3)	
4	26 (65)	14 (35)		38 (95)	2 (5)	
Number of parathyroids auto-transplanted						
0	193 (81.8)	43 (18.2)	**0.005** ^c^	212 (90.2)	23 (9.8)	0.262^c^
1	34 (66.7)	17 (33.3)		46 (90.2)	5 (9.8)	
2	3 (42.9)	4 (57.1)		5 (71.4)	2 (28.6)	

Values in parentheses are percentages

IQR = interquartile range

^a^Chi-squared test with Yates correction

^b^Mann–Whitney *U* test.

^c^Fisher’s exact test.

**Figure 1:**
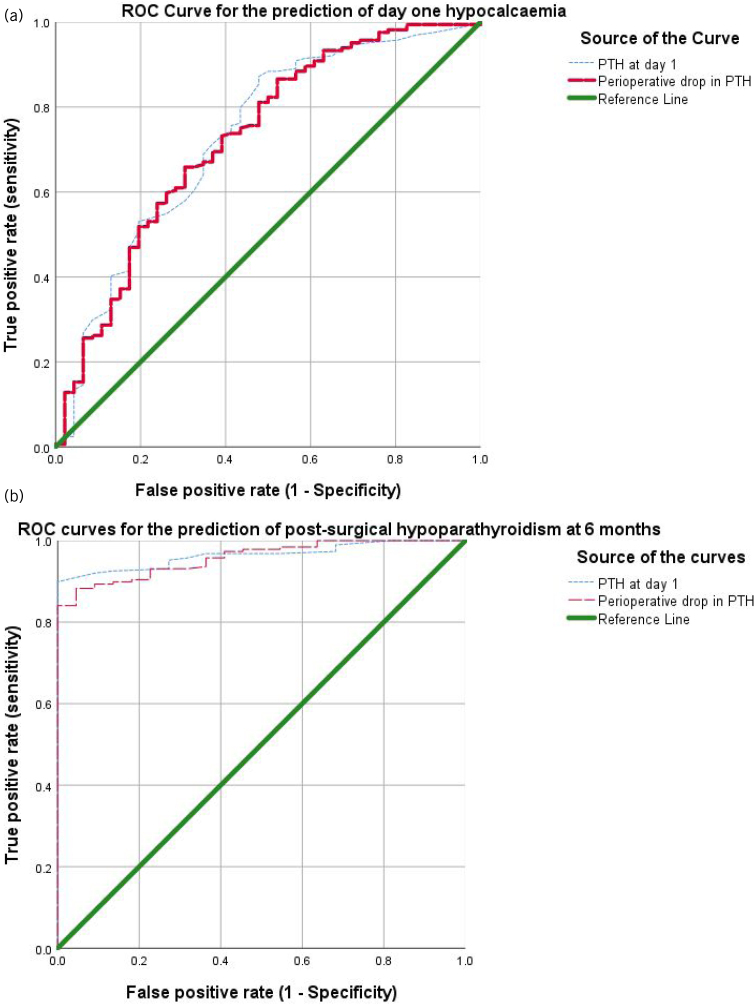
Receiver operating characteristic (ROC) curves demonstrating the value of postoperative parathyroid hormone (PTH) and perioperative PTH in predicting hypocalcaemia on the day after thyroid surgery (a), and postsurgical hypoparathyroidism at 6 months after surgery (b).

ROC curves were used to study the relationship between PTH levels, on the one hand, and transient hypocalcaemia and 6-month PoSH, on the other hand. For day 1 hypocalcaemia, the area under the curve (AUC) for day one PTH was 0.729, whereas that for drop in PTH was 0.726 (Figure 1a). For 6-month hypoparathyroidism, the AUC values were 0.964 and 0.958 respectively (Figure 1b).

## Discussion

Hypocalcaemia and/or hypoparathyroidism is a well-known complication of thyroid surgery. Early detection and appropriate treatment may help minimise patient morbidity and reduce hospital stay.^23,24^

This unit has previously reported its outcome relating to postsurgical hypoparathyroidism in two previous publications.^14,25^ The first report evaluated risk factors in detail, in addition to reporting on temporary and long-term PoSH rates, and suggested that day 1 calcium estimations alone may underreport the incidence of temporary hypocalcaemia.^25^ The second report detailed the development and validation of a protocol based on day 1 calcium and PTH estimations for the early detection and management of PoSH.^14^ This report presents the rates of temporary and long-term hypoparathyroidism after implementation of this protocol. Table 3 demonstrates outcomes relating to PoSH over the three study periods. Although day 1 hypocalcaemia in this recent study (21.7%) is lower compared with the reports in 2014 and 2018 (29.0% and 29.1%, respectively), the rates of patients requiring supplements were higher in this recent study (33.9%) compared with the previous two reports (18.4% and 15.9%, respectively). In addition, rates of long-term hypoparathyroidism were also higher in this study (10.5%) compared with the previous two reports (5.5% and 3.2%, respectively). Following implementation of the protocol, patients with low PTH but normal adjusted calcium levels on the day after surgery were initiated on treatment (which was not the practice previously). This raises the question as to whether adoption of a rigid protocol for early detection and treatment results in over-treatment and the lack of rigorous weaning strategies in patients started on supplementation. Another factor in the rate of long-term PoSH could have been the increase in the proportion of patients undergoing central neck dissection in this report (26.4%) compared with the previous two reports (21.1% and 22.3%, respectively).

**Table 3:** Comparison of outcomes relating to postsurgical hypoparathyroidism in the unit over three consecutive audits

Outcome	2008–2011 (*n* = 238)	2012–2014 (*n* = 282)	2016–2018 (*n* = 295)
Day 1 hypocalcaemia (adjusted calcium <2.1mmol/L)	69/238 (29.0)	82/282 (29.1)	64/295 (21.7)
Need for postoperative supplements	42/228 (18.4)	44/277 (15.9)	100/295 (33.9)
Need for supplements at 6 months	12/220 (5.5)	9/277 (3.2)	31/294 (10.5)

Values in parentheses are percentages

In a recent multicentre cohort study, a drop in PTH on the first postoperative day compared with the preoperative value was a reliable predictor of hypoparathyroidism.^16^

In this report, perioperative drop in PTH was not different from a single postoperative PTH value in its ability to predict transient hypocalcaemia and long-term hypoparathyroidism. The ROC curves (Figure 1a and 1b) demonstrate that the AUC for postoperative PTH was marginally better than the perioperative drop in PTH. This corroborates the results of a recent study which demonstrated that perioperative decline did not add much value to the diagnosis of PoSH compared with a single value of PTH measured 2h after thyroidectomy.^26^ In another study of 349 patients undergoing total thyroidectomy, a 70% decline in PTH at 1h after surgery had a sensitivity and specificity of predicting hypocalcaemia of 84.1% and 95%, respectively. However, in this study, 1-h PTH with a threshold of 10.42pg/ml was better than decline in PTH, with a sensitivity and specificity of 83.4% and 100%.^27^ It may be that the decline is not better than absolute values because hypocalcaemia may have causes other than hypoparathyroidism and this can in turn affect PTH decline.

Although it appears logical to assume that baseline PTH values and the decline in PTH after surgery may be expected to be a better predictor of PoSH compared with an absolute postoperative value, this study and others suggest that this is not the case. This means that measurement of PTH before surgery may not be of value, reducing the need for unnecessary PTH estimations before surgery.

### Study limitations

There are several limitations inherent in this observational study. The preoperative PTH were done at different time intervals. However, this is a pragmatic approach because in real life preoperative blood tests may be done at different times before the operation. The management of patients with PoSH was determined by the levels of adjusted calcium and PTH on the first postoperative day and not the drop in PTH values. This may have biased the comparison of the postoperative PTH and the decline in PTH in the prediction of long-term PoSH. The sample size of this cohort could be considered a limitation, particularly given the small number of patients with long-term hypoparathyroidism. However, many more patients had transient hypocalcaemia; given the similarity of results for both outcomes (transient hypocalcaemia and long-term PoSH), it would be reasonable to conclude that the drop in PTH is unlikely to be a significantly improved predictor of PoSH. Preoperative PTH may be influenced by several variables including vitamin D sufficiency and renal disease. The lack of adjusting for these conditions may also have biased the results.

In conclusion, this study shows that a single postoperative PTH value is sufficient in predicting both transient hypocalcaemia and postsurgical hypoparathyroidism. Measuring preoperative PTH values and estimating the decline in PTH does not add value to the prediction of postsurgical hypoparathyroidism.
